# Glucocorticoid‐mediated modulation of morphological changes associated with aging in microglia

**DOI:** 10.1111/acel.12790

**Published:** 2018-06-07

**Authors:** Lynn van Olst, Pascal Bielefeld, Carlos P. Fitzsimons, Helga E. de Vries, Marijn Schouten

**Affiliations:** ^1^ Department of Molecular Cell Biology and Immunology VU University Medical Center, Amsterdam Neuroscience Amsterdam The Netherlands; ^2^ Neuroscience Program Swammerdam Institute for Life Sciences University of Amsterdam Amsterdam The Netherlands

**Keywords:** glucocorticoid receptor, glucocorticoids, hippocampus, inflammaging, microglia, neuroimmunology, neuroinflammation

## Abstract

Microglia dynamically adapt their morphology and function during increasing age. However, the mechanisms behind these changes are to date poorly understood. Glucocorticoids (GCs) are long known and utilized for their immunomodulatory actions and endogenous GC levels are described to alter with advancing age. We here tested the hypothesis that age‐associated elevations in GC levels implicate microglia function and morphology. Our data indicate a decrease in microglial complexity and a concomitant increase in GC levels during aging. Interestingly, enhancing GC levels in young mice enhanced microglial ramifications, while the knockdown of the glucocorticoid receptor expression in old mice aggravated age‐associated microglial amoebification. These data suggest that GCs increase ramification of hippocampal microglia and may modulate age‐associated changes in microglial morphology.

## 1 INTRODUCTION

Microglia, the brain‐resident macrophages, are active players in the maintenance of neuronal networks (Wu, Dissing‐Olesen, MacVicar & Stevens, 2015). Microglia are long‐lived cells that are maintained and self‐renewing throughout life (Ajami, Bennett, Krieger, Tetzlaff & Rossi, 2007). Recent findings indicate that their renewal and age‐dependent transcriptome are highly dependent on their microenvironment and display region specificity within the CNS (Grabert et al., 2016; Tay et al., 2017). Notably, young hippocampal microglia were found to have a distinct and unique transcriptional phenotype that dissipated with increasing age (Grabert et al., 2016), suggesting that these microglia may respond distinctively to aging processes. However, how these age‐related differences arise and from what mediators they originate is still not fully understood.

Hormones are powerful chemical mediators that are able to transform a cell‐extrinsic signal into an altered cell function and gene expression profile. Aging is reported to provoke endocrine changes related to a plethora of pathophysiological conditions ranging from menopause to diabetes (Jones & Boelaert, 2014). Glucocorticoids (GCs) are a class of steroid hormones, which generally produce an anti‐inflammatory microglia profile (Bellavance & Rivest, 2014). The effects of GCs are mediated through the mineralocorticoid (MR) and glucocorticoid receptor (GR) (Reul & De Kloet, 1985), with the latter being more abundantly expressed in the microglia of young mice (Sierra, Gottfried‐Blackmore, Milner, McEwen & Bulloch, 2008). Both humans and rodents show age‐related changes in circulating GC plasma levels (Fitzsimons et al., 2016), possibly rendering aged microglia sensitive to their immunomodulatory effects.

To date, however, no literature has fully described how age‐related increases in GC impact hippocampal microglia function. As the microglial morphology is dynamically altered depending on its cellular function (Fernández‐Arjona, Grondona, Granados‐Durán, Fernández‐Llebrez & López‐Ávalos, 2017), we hypothesized that age‐associated increases in GC levels implicate hippocampal microglia cell morphology and therefore possibly their function. By both artificially increasing GC levels and using specific siRNAs to knockdown GR expression *in vivo*, we here describe a mechanism wherein GCs modulate hippocampal microglial morphology and may affect microglial amoebification during aging.

## 2 RESULTS/DISCUSSION

**Figure 1:**
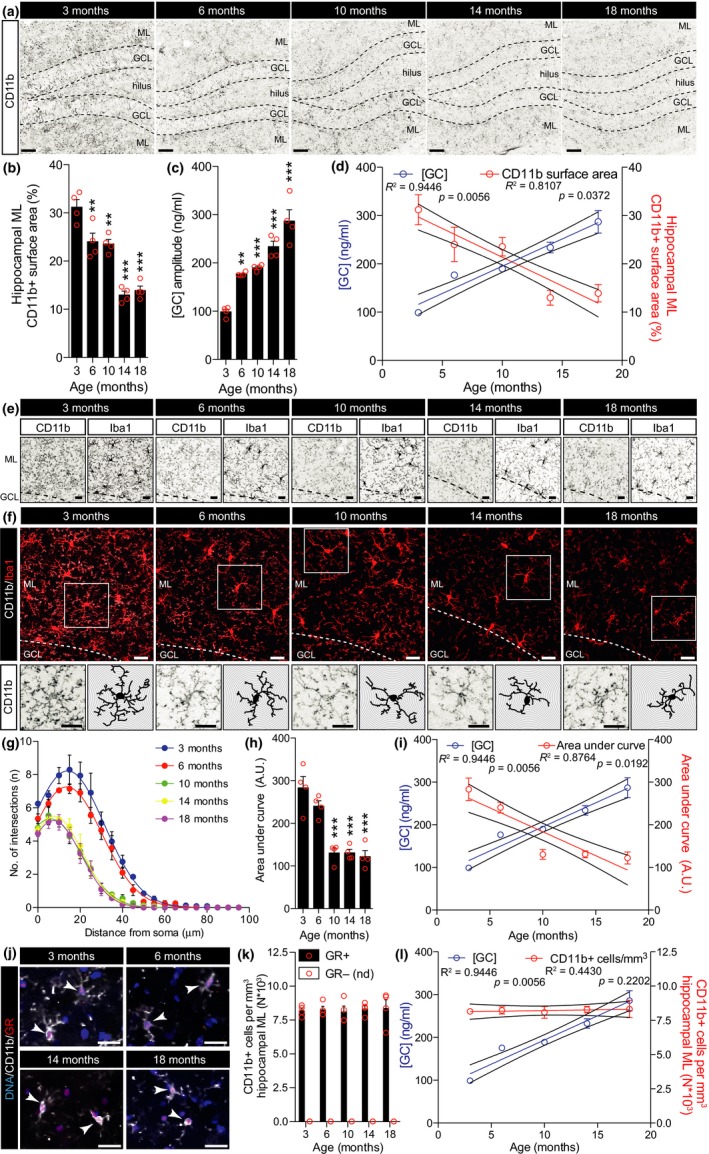
Age‐associated alterations in GC levels inversely correlate with age‐associated decreases in both CD11b+ cell coverage and morphological complexity. (a) Micrographs displaying hippocampal CD11b staining. (b) Hippocampal CD11b surface area percentage bar graph. (c) Blood plasma circadian amplitude [GC] bar graph. (d) Regression curves of plasma [GC] and hippocampal CD11b surface area. (e) Micrographs displaying hippocampal sections’ CD11b (left) and Iba1 (right) immunoreactivity. (f) Micrographs displaying hippocampal sections’ CD11b (white) and Iba1 (red) immunoreactivity. Boxed area (top) is magnified in bottom panels of individual CD11b+ cell morphology (left) and traces (right). (g) Sholl plots displaying CD11b+ cell branch intersections per 5 μm steps from the cell soma. (h) Sholl‐derived area under curve (arbitrary units: A.U.) bar graph. (i) Regression curves of plasma [GC] and hippocampal CD11b+ cell complexity as shown in Sholl‐derived area under curve (A.U.). (j) Single z‐plane confocal micrographs displaying CD11b+ cells with GR+ nuclei (arrowheads). (k) CD11b+/GR+ or GR‐ cell quantification (nd, not detected). (l) Regression curves of plasma [GC] and hippocampal CD11b+ cells/mm^3^. Significant differences are indicated as follows: **p <* *0.01, ***p <* *0.001, vs. 3 months, one‐way ANOVA. Scale bars = 50 (a), 20 (e,f), and 16 (j) μm.

**Figure 2:**
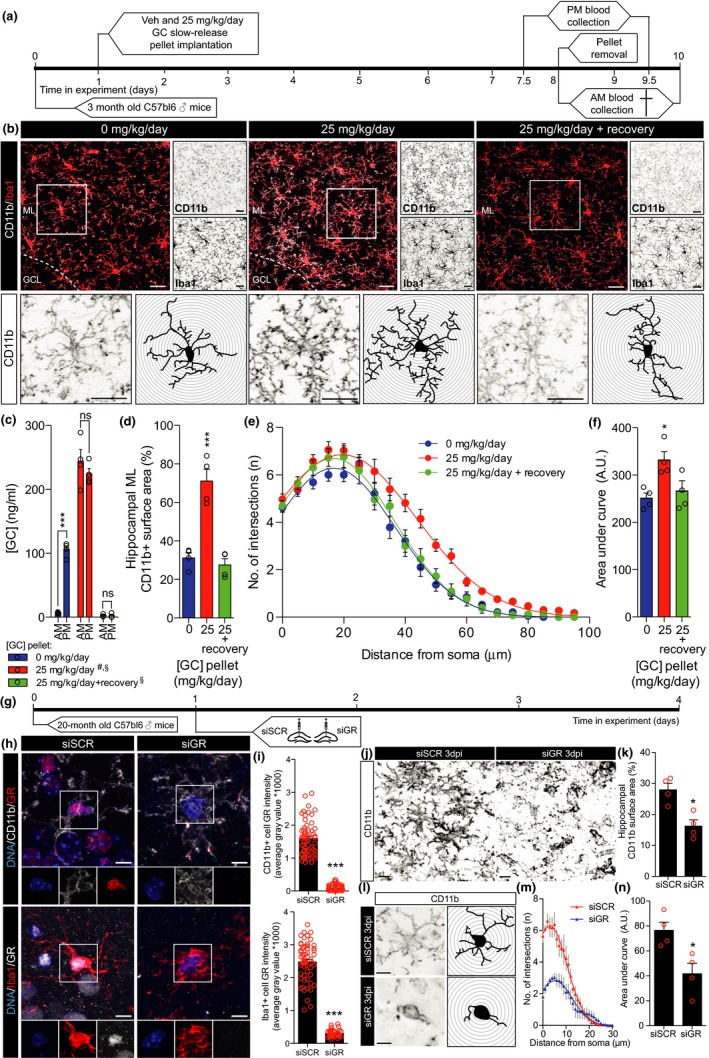
GC‐mediated modulation of hippocampal CD11b+ cell morphology in 3‐month‐old mice and GR‐mediated modulation of hippocampal CD11b+ cell morphology in 20‐month‐old mice. (a) Experimental setup used for panels b–f. (b) Micrographs displaying hippocampal CD11b (white) and Iba1 (red) staining after GC treatment on low magnification (top left) and separate immunoreactivity of CD11b and Iba1 (top right). CD11b staining in boxed area of the top left panel is magnified (bottom left panels) to show individual cells and their respective tracings (bottom right panels). (c) AM and PM blood plasma GC levels bar graph and significant differences are indicated as follows: ***p <* *0.001, AM vs. PM in vehicle, ns *p *>* *0.05, AM vs. PM in GC and GC + recovery; #*p *<* *0.001, CORT AM and PM vs. vehicle AM and PM; §*p *<* *0.001, both AM and PM in vehicle and GC vs. GC + recovery. (d) Hippocampal CD11b surface area bar graph. (e) Sholl plots displaying CD11b+ cell intersections per 5 μm steps from the cell soma. (f) Sholl analysis‐derived area under curve (arbitrary units: A.U.). (d–f) Significant differences are indicated as follows: *p <* *0.05, ***p <* *0.001, vs. vehicle, one‐way ANOVA. (g) Experimental setup used for panels h‐n. (h) Micrographs displaying GR intensity in CD11b+ (top panels) and Iba1+ (bottom panels) cells of siSCR (left)‐ and siGR (right)‐injected hippocampi. Boxed areas are shown as separate channels below. (i) CD11b+ (top) and Iba1+ (bottom) cell nuclear GR intensity quantifications for siSCR and siGR treatments. (j) Micrographs displaying CD11b staining of siSCR (left)‐ and siGR (right)‐treated hippocampi. (k) Hippocampal CD11b surface area quantification. (l) Micrographs displaying individual CD11b+ cell morphology (left) and traces (right) of siSCR (top)‐ and siGR (bottom)‐treated hippocampi. (m) Sholl plots displaying CD11b+ cell branch complexity per 1 μm steps from the cell soma. (n) Sholl analysis‐derived area under curve quantifications of siSCR‐ and siGR‐treated mice (arbitrary units: A.U.) (i, k, and n) Significant differences are indicated as follows: *p <* *0.05, ***p <* *0.001, siSCR vs. siGR, Student's *t* test. Scale bars = 20 (b), 10 (h), and 7 μm (j and l).

First, hippocampal microglia complexity was correlated to daily levels of [GC] (Figure 1a–d). Microglial coverage was assessed by a percentage of thresholded CD11b+ surface area in the molecular layer (ML) of the hippocampus and CD11b+ cell morphological complexity with Sholl analysis in the same region as previously described by Hoeijmakers et al. (2017). Both Iba1 and CD11b reliably stained microglia, yet side‐by‐side comparison of Iba1 and CD11b staining on the same cells in 6‐month‐old untreated mice revealed that CD11b stained a significantly higher amount of microglial ramifications (Supporting Information Figure S1). At 6 months of age, both hippocampal and cortical CD11b coverage started to decline (Figure 1a,b and Supporting Information Figure S2) coinciding with persistent increases in daily [GC] (Figure 1c). Combined, hippocampal CD11b coverage and [GC] both followed significant yet opposite trends during advancing age (Figure 1d). Extending these results, the relationship between both hippocampal and cortical CD11b surface area expression with age‐associated [GC] showed a significant inverse correlation (Supporting Information Figure S2e,f). Next to that, we found that the rate at which CD11b surface area expression decreased with age was significantly higher in the cortex compared to the hippocampus (Supporting Information Figure S2d), a result corroborating previous findings from Grabert et al. (2016) describing brain‐region‐dependent microglial aging. The age‐associated decreases in hippocampal and cortical CD11b surface area were phenocopied by the Iba1 costaining immunoreactivity (Figure 1e and Supporting Information Figure S2a). We next analyzed microglial complexity by means of CD11b+ cell Sholl analysis (Figure 1f). Sholl analysis supported that at 6 months, hippocampal microglia cell complexity declined (Figure 1f–h). Also, the CD11b+ cell complexity showed a significant inverse correlation with age and followed the opposite trend of [GC] (Figure 1i). We found CD11b+ cell numbers to remain stable with age, and we did not detect any CD11b+/GR‐ cells in these experimental conditions (Figure 1j–l). These data suggest that age‐associated GC elevations may associate with an amoeboid microglial phenotype. To selectively study the effect of increasing [GC] without other age‐related variables, [GC] was increased in 3‐month‐old mice using slow‐release GC pellets for the duration of 7 days. Mice were subsequently sacrificed either immediately or after a 2‐day recovery period following exogenous GC removal (Figure 2a), and from an CD11b/Iba1 costaining, hippocampal CD11b coverage and cell complexity were measured (Figure 2b). Surprisingly, increasing daily [GC] (Figure 2c) induced a significant increase in both hippocampal CD11b coverage (Figure 2d) and CD11b+ cell ramifications (Figure 2e,f) and these alterations were rapidly reversed during the 2‐day recovery (Figure 2b–f). To investigate whether [GC] could impact on measures indicative of microglial phagocytic activity, hippocampal CD68 surface area and CD68+ lysosomal volume of hippocampal Iba1+ cells were analyzed. However, the GC‐induced increases in ramifications were not accompanied by increases in either hippocampal CD68 expression, nor CD68+ lysosomal volume of hippocampal Iba1+ cells (Supporting Information Figure S3). In a primary attempt to exclude any possible indirect effect of GC treatment *in vivo*, we isolated human microglia to study the direct effects of GR modulation on microglial morphology *in vitro*. Our microglial isolation procedure yielded a >95% population of live CD45+/CD11b+ cells (Supporting Information Figure S4a) that robustly expressed Iba1 (Supporting Information Figure S4c). *In vitro*, Iba1+ cell surface area was significantly reduced after 72 hour treatment with dexamethasone (DEX; synthetic GR agonist), yet reversed by costimulation with both mifepristone (MIF; synthetic GR antagonist) and DEX (Supporting Information Figure S4d). Interestingly, the DEX‐induced reduction in microglial cell size was predominantly originated from a reduced soma size (Supporting Information Figure S4), as it also coincided with the extension of microglial processes (Supporting Information Figure S4f). Importantly, antagonizing DEX‐mediated GR activation using MIF reversed these morphological alterations, *in vitro* (Supporting Information Figure S4d–f). That GCs induced extension of microglial processes *in vitro* and a ramified microglial morphology *in vivo* could suggest that elevated [GC] induce a homeostatic microglial phenotype in the young mice. Therefore, we hypothesized that reducing microglial sensitivity to GC in old mice should further aggravate age‐related microglial amoebification. To test this hypothesis, GR expression was knocked down in 20‐month‐old mice (Figure 2g) as previously described (Fitzsimons et al., 2013; Schouten et al., 2015). Of note, limitations of this siRNA‐mediated knockdown approach include the intrahippocampal delivery itself that might induce alterations in the tissue for which we controlled with noncoding siRNA and the lack of cell‐type specificity. The latter might be accompanied by neuronal GR knockdown impacting spine density (Fitzsimons et al., 2013), possibly contributing to changes in microglial morphology. Nevertheless, nuclear GR protein levels in CD11b+/Iba1+ cells were reduced to ~10%, 3 days post‐injection (dpi; Figure 2h–i), which was accompanied by a decrease in hippocampal CD11b coverage (Figure 2j,k) and microglial complexity (Figure 2l–n).

Amongst the many hallmarks of aging (López‐Otín, Blasco, Partridge, Serrano & Kroemer, 2013) that may impact on microglia, our data indicate that increases in [GC] enhance microglial ramification, which may modulate age‐associated microglial amoebification. Further research should point out which other age‐related variables contribute to these morphological changes of microglia. Our work supports previous evidence describing dystrophic microglial in the aged brain (Streit, Sammons, Kuhns & Sparks, 2004), age‐related microglial changes in actin (dis)assembly genes that arrange the cell cytoskeleton (Galatro et al., 2017), and a decline in engagement of microglia with their environment in a brain‐region‐dependent manner (Grabert et al., 2016). Interfering with these processes by GC‐mediated modulation of neuroinflammation under (patho)physiological conditions might help to restore microglial homeostasis during aging and therefore possibly neuronal network maintenance as well as behavior (Bilbo & Schwarz, 2012).

## AUTHOR'S CONTRIBUTION

LvO, PB, and MS performed experiments and analyzed data; CPF and HEV participated in experimental design, result discussion, interpretation, and manuscript preparation. LvO, HEV, and MS conceived the study, designed experiments, analyzed and interpreted results, and wrote the manuscript.
