# Supplementary figures and images for: Glucocorticoid‐mediated modulation of morphological changes associated with aging in microglia
Source: Aging Cell. 2018 Jun 7;17(4):e12790. doi: 10.1111/acel.12790 (PMC6052476; doi:10.1111/acel.12790)

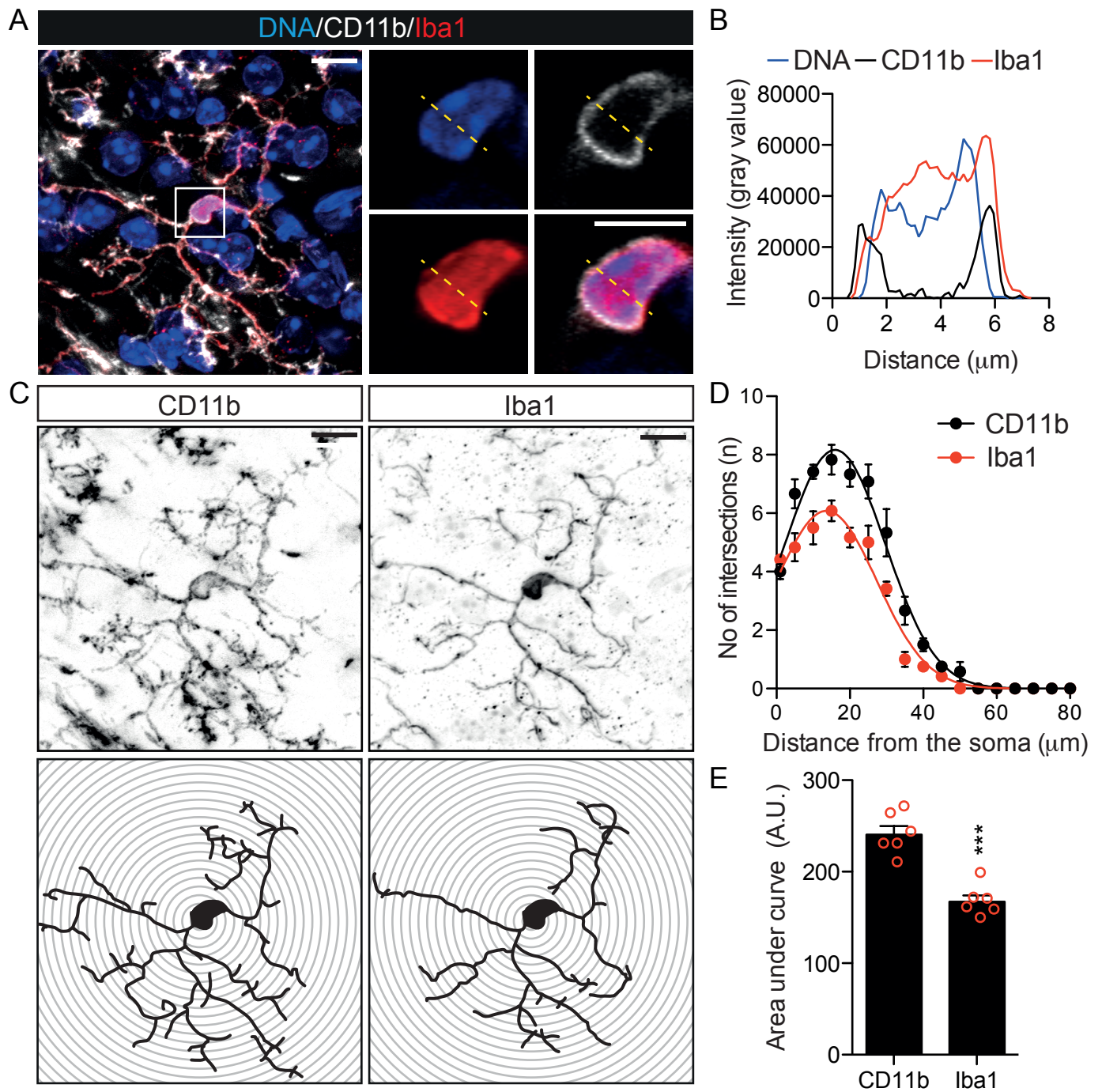

Supplement: Supplementary file 1 [file ACEL-17-na-s001.pdf]

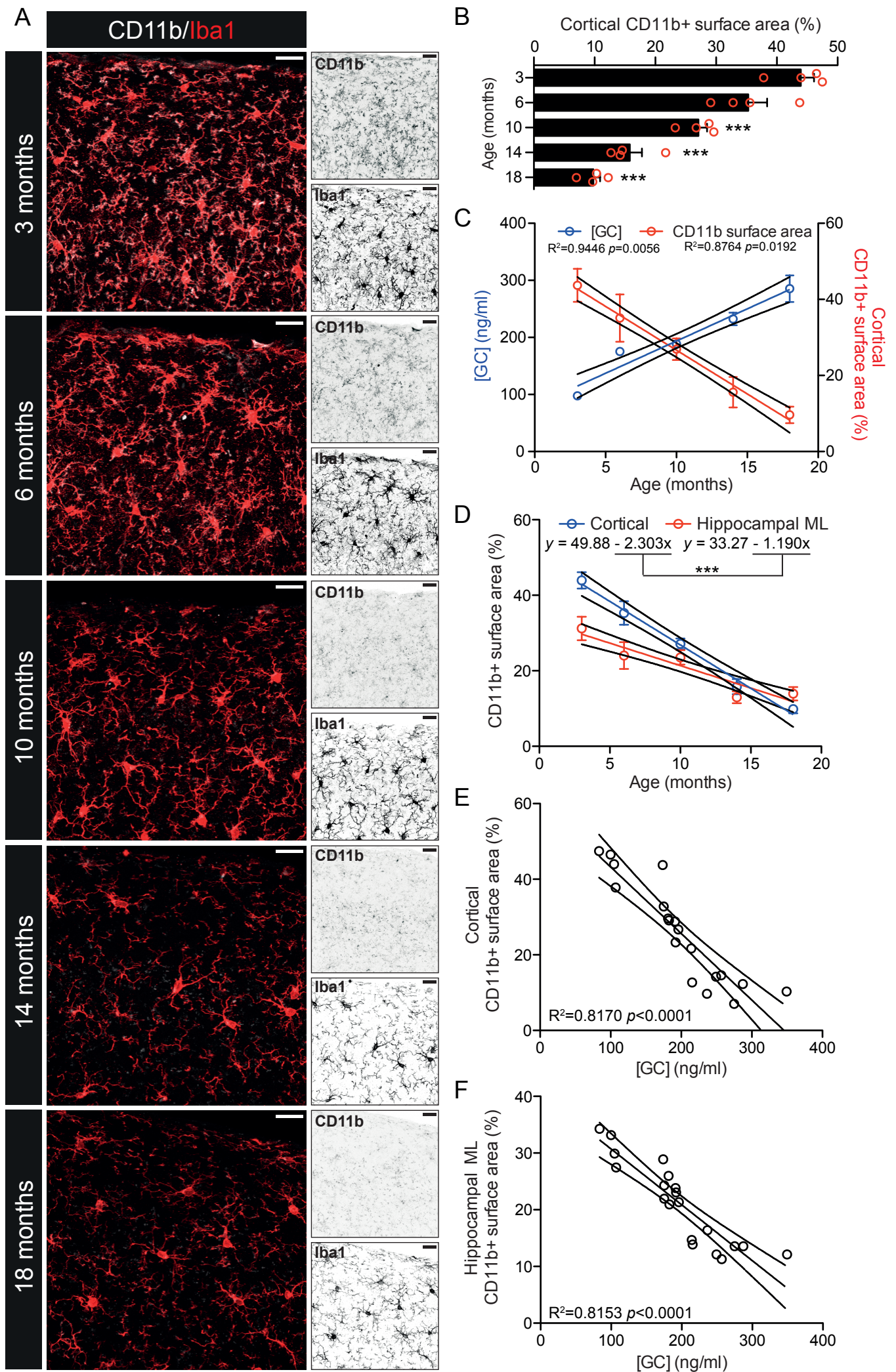

Supplement: Supplementary file 2 [file ACEL-17-na-s002.pdf]

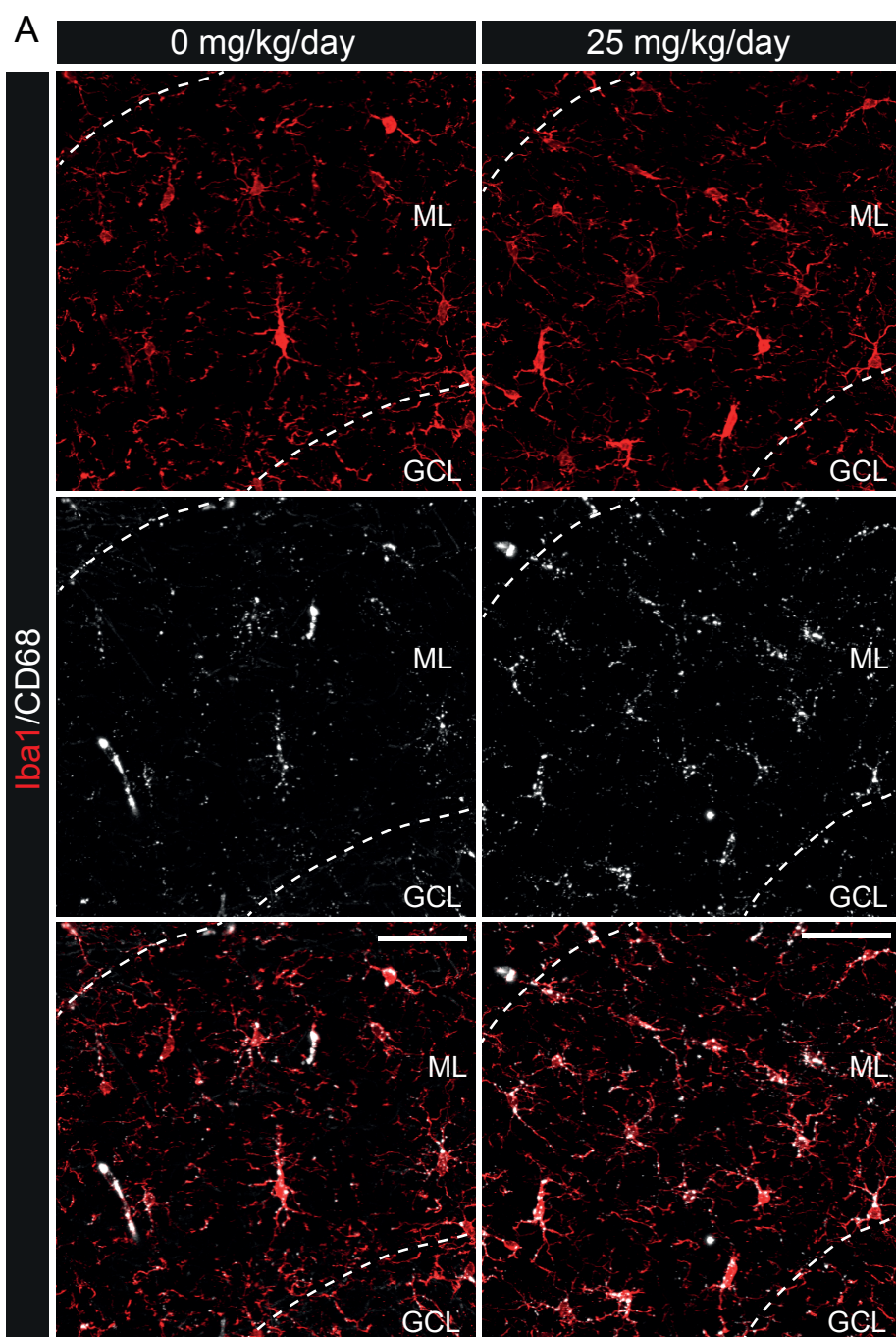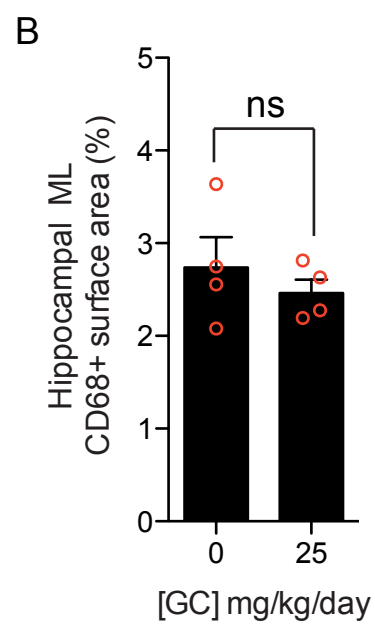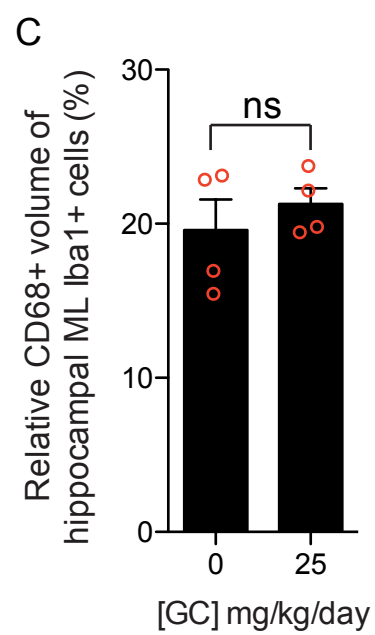

Supplement: Supplementary file 3 [file ACEL-17-na-s003.pdf]

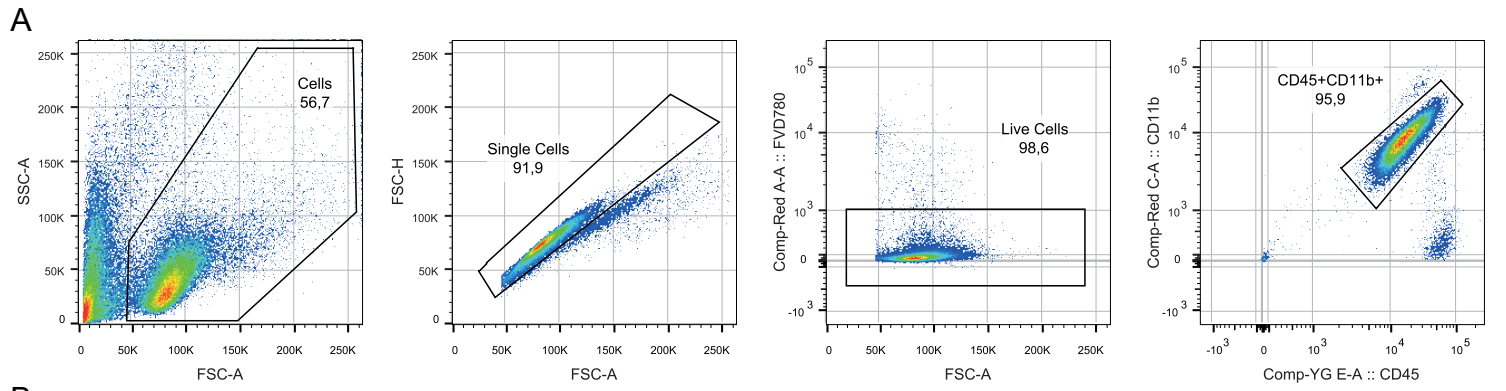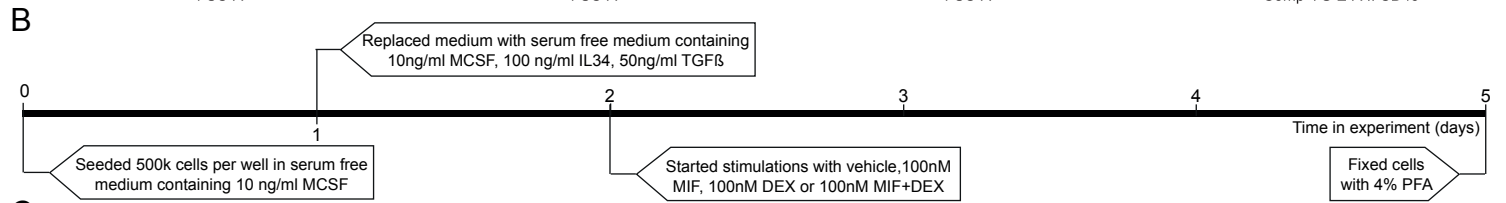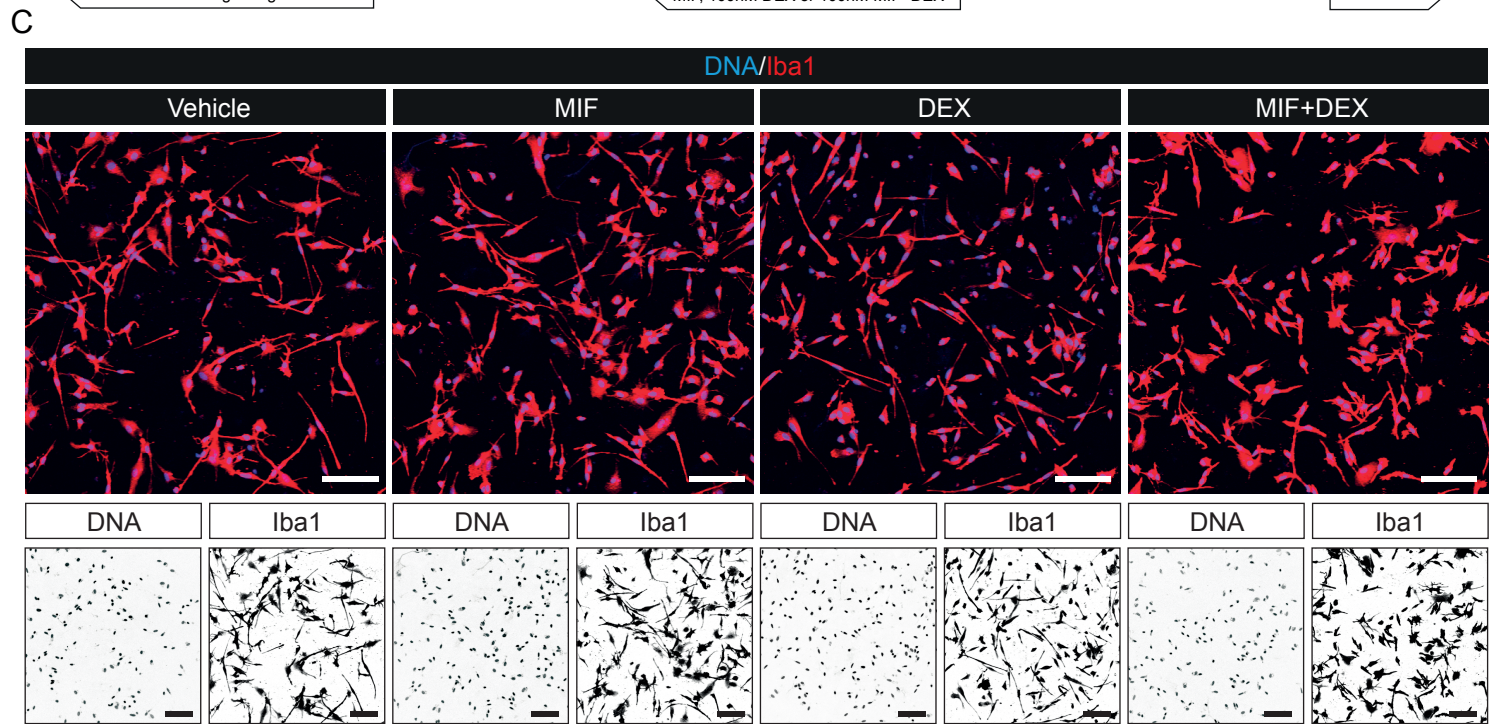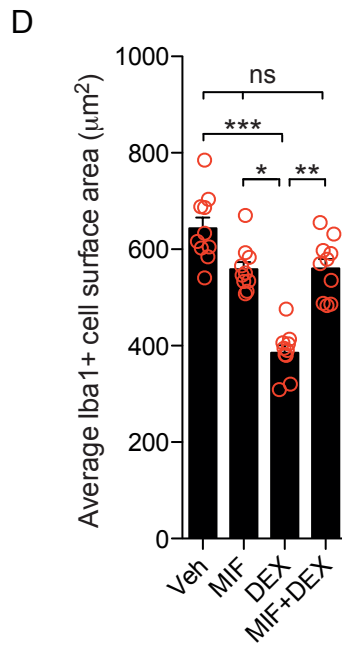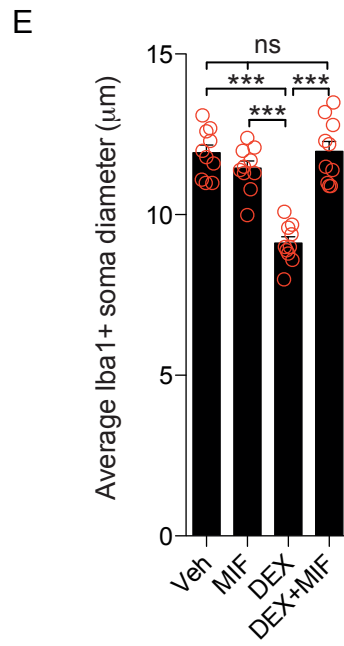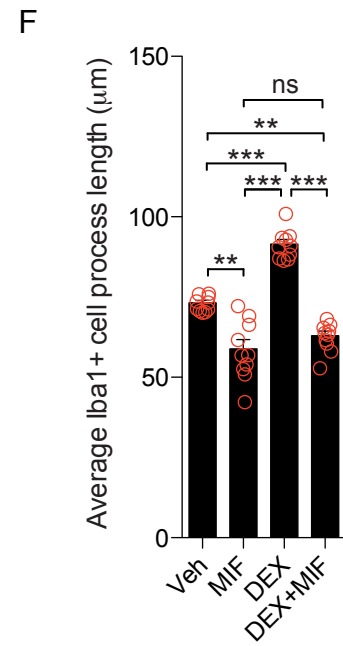

Supplement: Supplementary file 4 [file ACEL-17-na-s004.pdf]
