# Supplementary material for: Glucocorticoid‐mediated modulation of morphological changes associated with aging in microglia
Source: Aging Cell. 2018 Jun 7;17(4):e12790. doi: 10.1111/acel.12790 (PMC6052476; doi:10.1111/acel.12790)
Supplement: Supplementary file 5 [file ACEL-17-na-s005.docx]

**Online supporting information**

**Supplementary figure legends**

*Figure S1 - Characterization of CD11b and Iba1 as markers for microglial complexity.*

(A) Maximum projection confocal micrograph displaying a representative hippocampal CD11b+/Iba1+ microglia (left) with the boxed section magnified in single z-plane micrographs (right) of a 6 month old mouse. Dashed yellow lines in (A) show the direction of scanning for histogram (B).

(C) Micrographs displaying individual cell morphology (top) and traces (bottom) of CD11b (left) and Iba1 (right) signal of the same microglia.

(D) Sholl plots displaying CD11b+ and Iba1+ cell branch complexity per 5µm steps from the cell soma.

(E) Sholl analysis-derived area under curve quantifications of CD11b+ and Iba1+ signal from the same microglia (arbitrary units: A.U.). Significant differences are indicated ****p*<0.001, CD11b+ vs. Iba1+, Student’s *t*-test. Scale bars=10 µm (A and C).

*Figure S2 - Analysis of cortical CD11b cell coverage reveals an inverse correlative relationship with both age and GC levels.*

(A) Micrographs displaying cortical CD11b (white, top right) and Iba1 (red, bottom right co-staining.

(B) Cortical CD11b surface area percentage bar graph. Significant differences are indicated ****p*<0.001, vs. 3 months, one-way ANOVA.

(C) Regression curves of plasma [GC] age and cortical CD11b surface area.

(D) Linear regression curves and functions between age and CD11b surface area expression of the cortex (blue) and hippocampal ML (red). Significant differences in the slope (change in CD11b surface area per month; Cortex: -2.303% per month and hippocampus: -1.190% per month) as analyzed by extra-sum-of-squares *F*-test are indicated ****p*<0.001.

(E) Regression curve of plasma [GC] and cortical CD11b surface area.

(F) Regression curve of plasma [GC] and hippocampal CD11b surface area.

Scale bars=20 µm (A).

*Figure S3 - Analysis of hippocampal CD68 expression after GC pellet treatment of 3 month old mice.*

(A) Maximum projection confocal micrographs displaying hippocampal Iba1 and CD68 staining after GC treatment.

(B) Hippocampal ML CD68 surface area bar graph.

(C) Relative CD68+ volume of hippocampal ML Iba1+ cells bar graph.

No significant differences were found (ns; *p*>0.05), Student’s *t*-test. Scale bars=20 µm (A).

*Figure S4 - Human microglial morphology analyses after pharmacological GR activity modulation.*

(A) FACS plots displaying the purity (>95% of all single, alive cells) of isolated primary human CD11b+/CD45+ myeloid cells after Percoll density separation used for the *in vitro* experiment of B-F.

(B) Experimental setup used for panels C-F.

(C) Micrographs displaying nuclear (blue, bottom left) and Iba1 (red, bottom right) staining after a 72h GR modulation, *in vitro*. Scale bars=100 µm.

(D) Bar graph displaying the average Iba1+ cell surface areas after a 72h GR modulation, *in vitro*.

(E) Bar graph displaying the average Iba1+ cell soma diameter after a 72h GR modulation, *in vitro*.

(F) Bar graph displaying the average Iba1+ cell process lengths after a 72h GR modulation, *in vitro*. Significant differences between experimental groups are indicated **p*<0.05, ***p*<0.01, ****p*<0.001,

**Experimental procedures**

*Aging mice, GC measurements, tissue processing and immunohistochemistry*

Ages of brain tissue and blood plasma collection were chosen based on a previously established continuum of murine life stages, covering mature adulthood (3-6 months), middle age (10-14 months) and finally old age (18+ months) (Flurkey et al., 2007). Accordingly, male 3, 6, 10, 14 and 18 month-old C57bl6 mice (n=4 per group) were used for experiments. The animals were kept in standard laboratory cages with ad libitum access to food and water and in 12 hour light/dark intervals (lights on at 08:00, lights off at 20:00). Tail cut derived blood was captured in chilled EDTA-coated tubes (Sarstedt, Etten-leur, The Netherlands) in a stress-free manner starting at 20:00 the night before and starting at 08:00 on the morning of perfusion, as previously described (Fluttert, Dalm, & Oitzl, 2000). Blood samples were subsequently centrifuged for 15 minutes at 13 krpm. The supernatant blood plasma was stored at −20°C. Accordingly, AM and PM plasma GC levels were measured using a radioimmunoassay (MP Biomedicals, Eindhoven, The Netherlands). Concerning brain tissue preparation, at the indicated ages mice were transcardially perfused with saline and 4% paraformaldehyde in PBS at 08:00±0.3hours. After the perfused brains were extracted, they underwent a post-fixation treatment overnight submerged in 4% paraformaldehyde in PBS. Subsequently, 8 series of 40µm brainsections were collected, interspacing the brain slices of each series by at least 250 µm. For immunohistochemistry the following antibodies combinations were used: monoclonal rat anti-CD11b (M1/70 Ebioscience, 1:500) or monoclonal rat anti-CD68 (FA-11 Serotec, 1:400) and polyclonal rabbit anti-GR (H300 Santa Cruz, 1:100) or polyclonal rabbit anti-Iba1 (019-19741 Wako, 1:1000) in combination with goat anti-rat Alexa647 (Invitrogen, 1:500) and goat anti-rabbit Alexa555 (Invitrogen, 1:500), respectively. Alternatively, to quantify GR immunoreactivity in Iba1+ cells, polyclonal rabbit anti-GR (H300 Santa Cruz, 1:100) and polyclonal goat anti-Iba1 (ab5076 Abcam, 1:500) in combination with donkey anti-rabbit Alexa647 (Invitrogen, 1:500) and donkey anti-goat Alexa555 (Invitrogen, 1:500) were used, respectively. After 1 hour blocking with blocking mix (5% serum and 0.5% triton in PBS), primary antibodies were incubated for 2 hours at room temperature, than overnight at 4°C in the same blocking mix. Secondary antibodies, dissolved in PBS, were incubated for 2 hours at room temperature. For assessing microglial GR knockdown after siRNA injections, brain slices were incubated with primary antibodies for 4 hours at room temperature, than 36 hours 4°C and secondary antibodies 4 hours at room temperature. Sections were counterstained for DNA using DAPI (Invitrogen. 1:10000) to detect DNA in cell nuclei.

*GC pellet placement experiments and blood plasma collection*

Slow release pellets were used to manipulate GC levels (vehicle and 25 mg/kg/day, n=4 per experimental group; Innovative Research of America), using a modified version of a previously established protocol (Sarabdjitsingh et al., 2010). Under isoflurane anesthesia, pellets were inserted subcutaneously between the shoulder blades and closed by suturing the skin of 3 month old male C57bl6 mice starting at 08:00h on day 1. On day 8, the vehicle and 25 mg/kg/day [GC] groups of animals were sacrificed as described above. For the recovery group, pellets were removed at 08:00h on day 8, and animals were allowed to recover for 2 days before being sacrificed as described above. Blood plasma was collected on day 7/8 (PM/AM, respectively) or on day 9/10 (PM/AM, respectively) 2 days after pellet removal (recovery group) see also Fig. 2A for experimental timeline. Subsequent immunohistochemitry and [GC] analyses were performed as described in the corresponding section.

*Intracranial siRNA injections*

In the GR knockdown experiments, intracranial infusions were performed in 20 month old male C57bl6 hemispheres (n=4 per experimental group) with 1µl of a 40µM mixture of either 4 previously validated (Fitzsimons et al., 2013) siRNAs (FlexiTube GeneSolution, Qiagen, CAGACTCAGCATGGAGAATTA, AAGCGTGATGGACTTGTATAA, CAGTGGTGCGATAGCAACAAA, AAGGAAGGTCTGAAGAGCCAA) against the mouse GR (Nr3c1, Entrez gene ID: 14815) into the left hippocampus or negative control siRNA (Qiagen: AATTCTCCGAACGTGTCACGT) into the contralateral hippocampus (anterior-posterior: -2.0, medial-lateral: ± 1.5, dorsalventral: -2.0) as previously described (Schouten et al., 2015). 3 days post-injection, the mice were perfused and their brains were extracted and processed for immunohistochemistry, as described above. See also (Fig. 2G)

*Microscopy and microglial coverage, complexity, cell number and GR expression quantifications.*

Per animal 4 immunostained brain sections were imaged using either a Zeiss imager D2 fluorescent microscope or a Nikon A1R confocal microscope. For our analyses the hippocampal molecular layer (ML) of the dentate gyrus or cortex were analyzed. The images also highlight the hilus and granule cell layer (GCL) of the dentate gyrus. For microglial coverage the thresholded CD11b signal was measured in the hippocampal ML or cortex using ImageJ and expressed as a relative surface area percentage of total surface. Per animal a minimum of 20 CD11b+ and Iba1+ cells of the hippocampal ML were morphologically analyzed using a Sholl analysis as a means for their complexity. In brief, CD11b+ and Iba1+ cell branches were traced and of these traces, branch intersections with concentric circles around the soma ware counted using a Sholl analysis plugin in ImageJ (Hoeijmakers et al., 2017). The analyzed cells were averaged per animal and plotted into Sholl curves from which the area under the curve was extracted in which higher area under the curve values reflect a higher complexity. CD11b+/GR+ and CD11b+/GR- cell numbers were manually counted. A CD11b+ cell was considered GR+ using as inclusion criteria a GR gray value signal of ≥200 (background) across the DAPI+ nucleus within a CD11b+ cell signal. CD11b+/GR+ and CD11b+/GR- cell numbers were expressed as numbers per ML volume (cells/mm^3^). To analyze GR knockdown efficiency *in vivo* the average GR signal across the DAPI+ nucleus of CD11b+ cells was measured using histograms in ImageJ and expressed as average nuclear GR gray value per cell.

*Human brain tissue*

Post-mortem brain tissue was provided by the Netherlands Brain Bank. Informed consent was obtained for brain autopsy and for the use of tissue and clinical information for research purposes. At autopsy, with a post-mortem delay of 6:33 hours, subcortical white matter from a 91-old donor was dissected and stored in Hibernate A medium (ThermoFisher Scientific) at 4°C and processed within 24 hours.

*Human microglial isolation procedure*

The collected brain tissue was dissected into small pieces using surgical blades (Swann Morton) and enzymatically digested in PBS containing 2 U/mL Liberase TL (Roche) and 33 ug/mL DNAse I (Roche) for 60 min at 37°C in 50 mL polypropylene tubes (Corning). In this time, the suspension was mixed every 15 min. To stop the enzymatic digestion, ice-cold RPMI containing 10 % FBS (Biowest, South American Origin), 10 mM EDTA (Gibco), 20 mM HEPES (Gibco) and 50 uM 2-mercapthoethanol (Merck) was added to the sample and the suspension was stirred for 10 min at 4°C. Then, the sample was diluted with RPMI 1640 medium (Gibco) containing 10% FBS, 1% penicillin/streptomycin (P/S, Lonza) and 1% glutamine (Lonza) and the suspension was filtered using a 70 um nylon filter (Falcon). The suspension was centrifuged at 1500 RPM for 7 min with deceleration 5 and the pellet was resuspended in 70% isotonic Percoll (GE Healthcare) and 37% isotonic Percoll was added carefully on top of the suspension. The suspension was centrifuged at 900g for 30 min with slow acceleration and deceleration. The upper myelin layer was removed and the mononuclear cells were collected from the interface of the two Percoll gradients. Purity of the microglia cells was determined by flow cytometry (BD LSR Fortessa X-20) by staining of the cells for 30 min on ice using directly labelled antibodies CD11b-APC (M1/70; Biolegend, 1:100), CD45-PE (5B1, Miltenyi Biotec, 1:30) and Fixable Viability Dye eFluor 780 (FVD780, eBioscience, 1:1000), followed by a fixation with 1% PFA (ThermoFisher Scientific) for 1h at 4°C.

The cells were plated at a density of 500K cells per well in 24 well plates containing cover glasses (VWR) coated with 0.1 mg/mL Poly-L-Lysine (Merck) in 300 uL of DMEM/F-12 medium (Gibco) containing 1% P/S and 1% glutamine but no FBS. 10 ng/mL human recombinant M-CSF (eBiosciences) was added immediately after plating. The next day, the cells were washed, the medium was refreshed and 10 ng/mL human recombinant M-CSF (eBiosciences), 100 ng/mL human recombinant IL-34 (Miltenyi Biotec) and 50 ng/mL human recombinant TGFβ (R&D systems) were added to the medium. Stimulations with 100 nM dexamethasone (Sigma), 100 nM mifepristone (Sigma) or vehicle only were performed at the second day after plating and lasted for 72h. After a total culture time of 5 days, the cells were fixed using 4% PFA and stained as described above.

*Statistical analyses*

All data reflect mean±SEM and all comparisons were statistically tested in GraphPad Prism 5.0 using either unpaired two-tailed Student’s *t*-tests for comparing two experimental groups, or one-way analysis of variance (ANOVA) test with Tukey’s post hoc tests when more than two groups were compared. With these statistical tests significant differences are indicated and reflect the following *p*-values: **p*<0.05, ***p*<0.01 and ****p*<0.001. Linear regression curves are depicted as mean±95% confidence intervals, Pearson correlation analyses from which R^2^ and significance *p*-values are indicated in the figures. An extra-sum-of-squares *F*-test was used for statistical comparisons between cortical and hippocampal age dependent loss of CD11b surface area percentage (slope of linear regression). With this statistical test significant differences are indicated and reflect the following *p*-values: ****p*<0.001 (Figure S2D). Sholl analysis curves from which area under the curve data were deduced were created and analyzed using Graphpad Prism 5.0.

**References**

Fitzsimons, C. P., van Hooijdonk, L. W. A., Schouten, M., Zalachoras, I., Brinks, V., Zheng, T., … Vreugdenhil, E. (2013). Knockdown of the glucocorticoid receptor alters functional integration of newborn neurons in the adult hippocampus and impairs fear-motivated behavior. *Molecular Psychiatry*, *18*(9), 993–1005. https://doi.org/10.1038/mp.2012.123

Flurkey, K; Currer, J M.; and Harrison, D E. (2007).*"Mouse models in aging research."* Faculty Research 2000 - 2009. 1685.

Fluttert, M., Dalm, S., & Oitzl, M. S. (2000). A refined method for sequential blood sampling by tail incision in rats. *Laboratory Animals*, *34*(4), 372–378. https://doi.org/10.1258/002367700780387714

Hoeijmakers, L., Ruigrok, S. R., Amelianchik, A., Ivan, D., van Dam, A.-M., Lucassen, P. J., & Korosi, A. (2017). Early-life stress lastingly alters the neuroinflammatory response to amyloid pathology in an Alzheimer’s disease mouse model. *Brain, Behavior, and Immunity*, *63*, 160–175. https://doi.org/10.1016/j.bbi.2016.12.023

Sarabdjitsingh, R. A., Isenia, S., Polman, A., Mijalkovic, J., Lachize, S., Datson, N., … Meijer, O. C. (2010). Disrupted Corticosterone Pulsatile Patterns Attenuate Responsiveness to Glucocorticoid Signaling in Rat Brain. *Endocrinology*, *151*(3), 1177–1186. https://doi.org/10.1210/en.2009-1119

Schouten, M., Fratantoni, S. A., Hubens, C. J., Piersma, S. R., Pham, T. V., Bielefeld, P., … Fitzsimons, C. P. (2015). MicroRNA-124 and -137 cooperativity controls caspase-3 activity through BCL2L13 in hippocampal neural stem cells. *Scientific Reports*, *5*(1), 12448. https://doi.org/10.1038/srep12448
